# Haploinsufficiency of autism causative gene *Tbr1* impairs olfactory discrimination and neuronal activation of the olfactory system in mice

**DOI:** 10.1186/s13229-019-0257-5

**Published:** 2019-02-11

**Authors:** Tzyy-Nan Huang, Tzu-Li Yen, Lily R. Qiu, Hsiu-Chun Chuang, Jason P. Lerch, Yi-Ping Hsueh

**Affiliations:** 1 Institute of Molecular Biology, Academia Sinica, 128, Academia Rd., Sec. 2, Taipei, 11529 Taiwan; 2 Mouse Imaging Centre, Hospital for Sick Children, Toronto, Canada; 3 Department of Medical Biophysics, The University of Toronto, Toronto, Canada; 4 Present address: Department of Molecular and Cell Biology, University of California, Berkeley, Berkeley, CA 94720 USA

**Keywords:** Autism spectrum disorders, C-FOS, D-cycloserine, Neuronal activation, Olfactory bulb, Olfactory discrimination, T-brain-1

## Abstract

**Background:**

Autism spectrum disorders (ASD) exhibit two clusters of core symptoms, i.e., social and communication impairment, and repetitive behaviors and sensory abnormalities. Our previous study demonstrated that TBR1, a causative gene of ASD, controls axonal projection and neuronal activation of amygdala and regulates social interaction and vocal communication in a mouse model. Behavioral defects caused by *Tbr1* haploinsufficiency can be ameliorated by increasing neural activity via D-cycloserine treatment, an N-methyl-D-aspartate receptor (NMDAR) coagonist. In this report, we investigate the role of TBR1 in regulating olfaction and test whether D-cycloserine can also improve olfactory defects in *Tbr1* mutant mice.

**Methods:**

We used *Tbr1*^*+/−*^ mice as a model to investigate the function of TBR1 in olfactory sensation and discrimination of non-social odors. We employed a behavioral assay to characterize the olfactory defects of *Tbr1*^*+/−*^ mice. Magnetic resonance imaging (MRI) and histological analysis were applied to characterize anatomical features. Immunostaining was performed to further analyze differences in expression of TBR1 subfamily members (namely TBR1, TBR2, and TBX21), interneuron populations, and dendritic abnormalities in olfactory bulbs. Finally, C-FOS staining was used to monitor neuronal activation of the olfactory system upon odor stimulation.

**Results:**

*Tbr1*^*+/−*^ mice exhibited smaller olfactory bulbs and anterior commissures, reduced interneuron populations, and an abnormal dendritic morphology of mitral cells in the olfactory bulbs. *Tbr1* haploinsufficiency specifically impaired olfactory discrimination but not olfactory sensation. Neuronal activation upon odorant stimulation was reduced in the glomerular layer of *Tbr1*^*+/−*^ olfactory bulbs. Furthermore, although the sizes of piriform and perirhinal cortices were not affected by *Tbr1* deficiency, neuronal activation was reduced in these two cortical regions in response to odorant stimulation. These results suggest an impairment of neuronal activation in olfactory bulbs and defective connectivity from olfactory bulbs to the upper olfactory system in *Tbr1*^*+/−*^ mice. Systemic administration of D-cycloserine, an NMDAR co-agonist, ameliorated olfactory discrimination in *Tbr1*^*+/−*^ mice, suggesting that increased neuronal activity has a beneficial effect on *Tbr1* deficiency.

**Conclusions:**

*Tbr1* regulates neural circuits and activity in the olfactory system to control olfaction. *Tbr1*^*+/−*^ mice can serve as a suitable model for revealing how an autism causative gene controls neuronal circuits, neural activity, and autism-related behaviors.

**Electronic supplementary material:**

The online version of this article (10.1186/s13229-019-0257-5) contains supplementary material, which is available to authorized users.

## Background

Autism spectrum disorders (ASDs) are highly prevalent neurodevelopmental disorders [1, 2]. Patients with ASD feature two core behavioral symptoms, termed the ASD dyad; one is social and communication impairment, and the other is repetitive behaviors and sensory abnormalities [3, 4]. Many molecular etiological studies using animal models have been conducted to investigate social interaction, vocal communication, and repetitive behaviors [5–8]. Regarding sensory dysregulation, mouse models with *Mecp2* and *Fmr1* deficiencies have been used to study defects in tactile, visual, auditory, and olfactory responses [9–19]. However, there have been fewer investigations of sensory dysregulation in other ASD animal models exhibiting deficiencies in other ASD causative genes. It is also unclear if mouse models can reflect the diverse variations of sensory dysfunction in patients with ASD.

Based on human genetic studies using whole-exome sequencing analyses, the brain-specific T-box transcription factor gene *T-brain-1* (*TBR1*) is a causative gene of ASD [20–22]. De novo loss-of-function and missense mutations in one allele of *TBR1* are recurrently identified in patients with ASD [20–22]. Echoing the mutations identified in patients, *Tbr1*^*+/−*^ mice exhibit autism-like behaviors, including reduced social interaction, impaired learning and memory, and aberrant cognitive flexibility [23].

*Tbr1* is critical for both forebrain development and neuronal activation. Deletion of *Tbr1* impairs neuronal migration of the cerebral cortex and amygdalae [24, 25], axonal projection of the cerebral cortex and amygdalae [23, 24], and differentiation of projection neurons in the olfactory bulb [26], resulting in neonatal lethality within 1–2 days of birth [26]. When only one of the two *Tbr1* alleles is deleted in mutant mouse models—representing a scenario imitating the genotype of ASD patients [20–22]—the gross anatomy and structure of the *Tbr1*^*+/−*^ mutant mouse brains do not exhibit obvious defects [23], but the posterior part of their anterior commissure (the white matter structure connecting the two amygdalae of the two brain hemispheres) is much smaller or even missing [23]. For amygdalar neurons, *Tbr1* heterozygosity influences the expression of a set of genes, including *Ntng1*, *Cntn2*, and *Cdh8* [23, 27], that impairs axonal extension and differentiation, thereby resulting in reduced inter- and intra-amygdalar axonal connections [23]. In addition to controlling axonal projection, *Tbr1* is also required for neuronal activation. It acts as an immediate early gene to bind the promoter of *Grin2b* [28, 29] and regulate *Grin2b* expression in response to neuronal activation [30]. Since *Grin2b* encodes a critical subunit of N-methyl-D-aspartate receptor (NMDAR), an important glutamate receptor involved in learning/memory and a variety of neurological disorders including autism and schizophrenia [20, 31], TBR1 regulates neuronal activity and functions by controlling *Grin2b* expression. Thus, TBR1 plays dual roles in neurons, namely regulation of axonal projection and control of neuronal activation. The axonal projection controlled by TBR1 necessitates correct neural circuit formation. The cell-autonomous effect of TBR1 on the control of *Grin2b* expression thereby synergizes with TBR1-mediated regulation of axonal projection to control the activity of specific neural circuits. This scenario is supported by the observation that local infusion of D-cycloserine, an NMDAR coagonist, into amygdalae ameliorates the autism-like behaviors exhibited by *Tbr1*^*+/−*^ mice [23]. Although the developmental defects (axonal projection) cannot be rescued, increased neuronal activity at the adult stage is sufficient to ameliorate the behavioral defects caused by *Tbr1* haploinsufficiency.

Studies of *Tbr1*^*−/−*^ mice have shown that, apart from the cortex and amygdalae, *Tbr1* is also critical for the development of projection neurons in the olfactory bulb [26, 32, 33]. It would be intriguing to explore whether *Tbr1*^*+/−*^ mice also exhibit impaired olfaction because olfactory dysfunction has been reported in patients with ASD [34–42]. Several mouse studies have revealed the influence of olfactory responses in social interaction and ultrasonic vocalization [43–45]. However, abnormal olfactory responses in ASD patients are not necessarily related to social interaction. Children with ASD tend to exhibit strong food selectivity, which is at least partially due to abnormal olfactory responses [38, 46]. Olfactory responses to non-social odors remain rather unexplored in mouse models harboring ASD-linked mutations.

Depending on cohorts and experimental design, the ASD-linked impairments in olfactory responses to non-social odors are quite diverse [47, 48]. Hypo- or hyper-olfactory sensitivity and impaired odor identification are frequently observed in ASD patients [34–42]. Previous studies have indicated that patients with ASD exhibit normal food odor-sensing ability but have a lower discrimination score [34, 35, 39]. We were interested in investigating whether *Tbr1* haploinsufficiency results in abnormal olfactory processing in response to non-social odors. Using *Tbr1*^*+/−*^ mice, we found that olfactory discrimination is sensitive to *Tbr1* deficiency. This outcome is probably due to altered cell identity (including changes in the property of projection neurons and the number of interneurons) and abnormal neuronal circuits in the olfactory system, which consequently reduces neuronal activation in the olfactory system including in the glomerular layer of olfactory bulbs and piriform and perirhinal cortices of *Tbr1*^*+/−*^ mice. As found for other autism-like behaviors, systemic administration of D-cycloserine fully ameliorated the defect of olfactory discrimination we observed in *Tbr1*^*+/−*^ mice. Our data suggest that *Tbr1* haploinsufficiency alters neuronal circuits in the olfactory system and impairs olfactory discrimination of non-social odors, one of the core symptoms of ASD.

## Methods

### Experimental design

*Tbr1*^*+/−*^ mice were used to evaluate the role of *Tbr1* in olfaction. Olfactory sensation and discrimination of *Tbr1*^*+/−*^ mice were first investigated by behavioral assay. Histological analysis, magnetic resonance imaging (MRI), and immunostaining were then used to characterize the etiology of *Tbr1* deficiency in terms of olfactory responses.

### Animals

The *Tbr1*^*+/−*^ mice [26] were originally provided by Drs. R. F. Hevner (Department of Neurological Surgery, University of Washington, Seattle) and J. L. Rubenstein (Department of Psychiatry, University of California, San Francisco). These mice were maintained by backcrossing into a C57BL/6 background for over 30 generations and were housed in a facility at the Institute of Molecular Biology, Academia Sinica. Male *Tbr1*^*+/−*^ mice and wild-type littermates at 2–3 months of age were used for the behavioral assay to avoid variations due to the estrus cycle and age. A 12-h light/dark cycle (lights off at 20:00) was maintained in the test room. Food and water were accessed ad libitum. All of the animals were housed in mixed-genotype groups of 3–5 mice per cage and were subjected to experiments randomly without any specific selection criteria. All of the animal experiments were performed with the approval of the Academia Sinica Institutional Animal Care and Utilization Committee.

### Behavioral assay

**Fig. 1:**
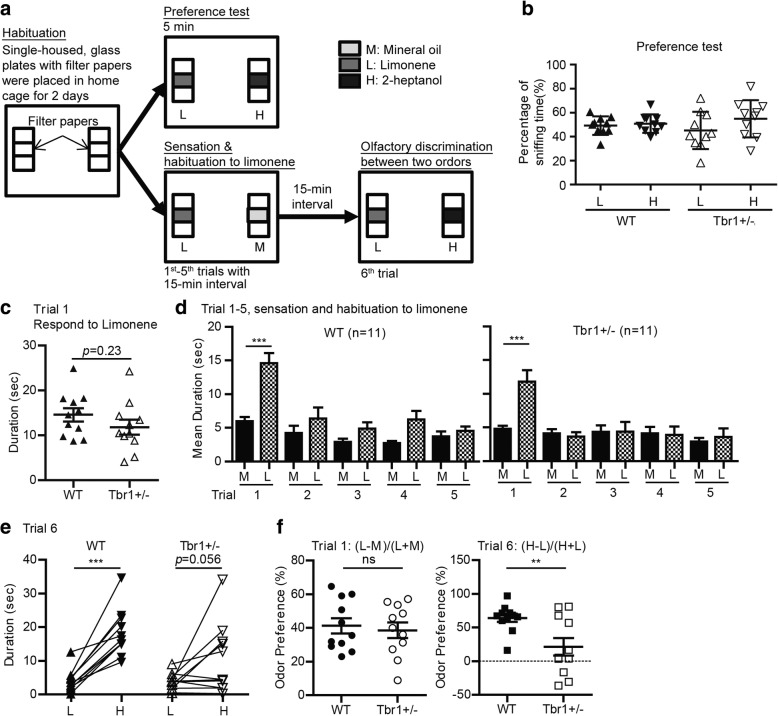
Olfactory discrimination deficiency in *Tbr*1^+/−^ mice. **a** Flow chart of our odor preference test (upper panel) and olfactory sensation-habituation-dishabituation test (lower panel). **b** The results of the preference test. Wild-type (WT) and *Tbr1*^*+/−*^ mice spend similar amounts of time sniffing limonene (L) and 2-heptanol (H). **c** The results of olfactory sensation. Sniffing time of limonene by WT and *Tbr1*^+/−^ mice is comparable in the first trial of our olfactory discrimination test. **d** The results of trials 1–5 of the sensation-habituation-dishabituation test. Olfactory habituation to limonene (L) is similar between WT and *Tbr1*^+/−^ mice. But *Tbr1*^+/−^ mice exhibit an olfactory discrimination deficiency in distinguishing 2-heptanol and limonene in trial 6. **e** Sniffing time of limonene (L) and 2-heptanol (H) by WT and *Tbr1*^+/−^ mice during trial 6. **f** Odor preference index from trials 1 and 6. The equations to calculate the odor preference index are indicated. Data are presented as mean plus SEM in (**b**), (**c**), (**d**), and (**f**). Data from individual mice are also indicated in (**b**), (**c**), (**e**), and (**f**). **p < 0.01; ***p < 0.001

Many behavioral features of *Tbr1*^*+/−*^ mice have been analyzed previously [23, 49]. The results of these studies indicated that *Tbr1*^*+/−*^ mice exhibit no obvious defect in locomotion, anxiety, novel object recognition, or contextual fear conditioning. However, mutant mice exhibit impaired amygdala-dependent associative memory, cognitive inflexibility, and reduced sociability. In this report, we focused on olfactory responses to non-social odorants, a feature of ASD. The experiments were conducted as described previously [50–52] with some modifications (Fig. 1a). Mice were individually housed during the entire experimental period. After being habituated to the experimental set-up outlined in (1) below, mice were separated into three different groups for the experiments described below in (2), (3), and (4). Data analysis was performed without knowing the genotype of the mice.

(1) Habituation to experimental setup (Fig. 1a, left). Two glass plates with filter papers were placed at the two ends of the home cage for 2 days. During this 2-day habituation, no odorant was added onto the filter papers.

(2) Preference test to investigate whether mice had any preference for specific odorants (Fig. 1a, upper). Limonene (Cat. No. 8.1840, Merck) and 2-heptanol (Cat. No. 8.20619, Merck), two very different odorants, were spotted individually onto filter papers placed at the two ends of the home cage of test mice. The sniffing behaviors of mice towards odorants were recorded using a camera installed on the lid of the cage. We measured the time taken to sniff the filter paper within 5 min of the start of the test. The percentage of sniffing time towards individual odorants over total sniffing time reflected whether mice had a preference for limonene or 2-heptanol (Fig. 1b).

(3) Six consecutive trials to test olfactory sensation (in trial 1), habituation (from trials 2–5), and dishabituation (trial 6) to specific odorants (Fig. 1a, lower). After confirming that mice had no preference for limonene or 2-heptanol, we used these two odorants, together with mineral oil as a control, to conduct the six consecutive trials. In the first five trials (5 min for each trial with 15-min intervals), limonene and mineral oil were individually spotted onto the filter papers placed at the two ends of the home cage of test mice. In the sixth trial, 2-heptanol was spotted onto the glass plate that originally held the filter paper spotted with mineral oil. Mouse behaviors during these six trials were recorded using a camera installed on the lid of the cage. The total time to sniff each filter paper was measured for each trial, which indicated the olfactory response to each odorant. The response to limonene in the first trial represented the “olfactory sensation” of mice. Repetitive exposures to limonene (trials 2 to 5) induced “habituation” and consequently reduced the amount of time mice spent sniffing limonene. The presence of 2-heptanol in trial 6 evoked “dishabituation” and resulted in more time spent sniffing 2-heptanol. Dishabituation induced by the secondary odorant (2-heptanol) indicates “olfactory discrimination”. In addition to assessing time spent sniffing each odorant, we also calculated odor preference indices. For trial 1, the index represented (sniffing time for limonene − sniffing time for mineral oil)/(sniffing time for limonene + sniffing time for mineral oil). For trial 6, the index was equal to (sniffing time for 2-heptanol − sniffing time for limonene)/(sniffing time for 2-heptanol + sniffing time for limonene).

(4) To examine the rescue effect of D-cycloserine, we intraperitoneally injected 20 mg/kg body weight of D-cycloserine into mice 30 min before the first of the six consecutive trials.

### Magnetic resonance imaging (MRI)

MRI imaging of mouse brains was performed as described [53]. Briefly, mice were anesthetized and intracardially perfused with 10 ml of 0.1 M PBS containing 10 U/ml heparin (PPC, cat#C504805) and 2 mM ProHance (Gadolinium contrast agent, Bracco Diagnostics, cat#111181) followed by 10 ml of 4% paraformaldehyde/PFA (Cedarlane cat#15710) containing 2 mM ProHance. After perfusion, mice were decapitated. The brain and remaining skull structures were incubated in 4% PFA with 2 mM ProHance overnight at 4 °C, then transferred to 0.1 M PBS containing 2 mM ProHance and 0.02% sodium azide for at least 7 days, before MRI scanning. The anatomical MRI scans used a T2-weighted, three-dimensional fast spin-echo sequence, with a cylindrical acquisition of k-space, and with a TR of 350 ms, TEs of 12 ms per echo for six echoes, a field of view of 20 × 20 × 25 mm^3^, and matrix size = 504 × 504 × 630 giving an image with 0.040-mm isotropic voxels. Total imaging time was ~ 14 h [54].

For the volume measurements, we computed the deformations needed to encompass the anatomy of each individual mouse in a common consensus space through iterative linear and nonlinear registrations, the goal being to model how the deformation fields relate to genotype. The Jacobian determinants of the deformation fields were then calculated as measures of volume at each voxel. Volume changes were additionally calculated by warping a pre-existing classified MRI atlas onto the population atlas, which allowed for the volume of 159 segmented structures encompassing cortical lobes, large white matter structures (i.e., corpus callosum), ventricles, cerebellum, brain stem, and olfactory bulbs to be assessed in all brains [55–57]. These measurements could then be examined on a voxel-wise basis to localize the differences found within regions or across the brain. Multiple comparisons were accounted for using the false discovery rate (FDR) [58].

### Immunohistochemistry

Mice were anesthetized and perfused with PBS, followed by 4% paraformaldehyde (PFA) in PBS. After postfixation with 4% PFA for 3–4 h at 4 °C, immersion in 30% sucrose for 2 days, and cryopreservation at − 80 °C, we collected coronal sections of 50 μm thickness. Brain sections were blocked with the blocking solution from the Tyramide Signal Amplification kit (ThermoFisher Scientific Inc.) for 1 h. Primary antibodies were then added and incubated overnight at 4 °C. The primary antibodies used in this report are as follows: rabbit anti-C-FOS (1:200; Cell Signaling), anti-calretinin (1:250; Swant), anti-calbidin (1:100; Cell Signaling), anti-neurofilament light chain (1:100; AB9568), anti-parvalbumin (1:200; Swant), rat anti-TBR2 (Eomes) (1:200; eBioscience 12-4875, PE-conjugated), anti-TBX21 (T-bet) (1:200; BD Biosciences), anti-VGLUT1 (1:200; Millipore AB5905), anti-VGLUT2 (1:200; Neuromab, 75-067). After washing, secondary antibodies conjugated with streptavidin, Alexa flour-488, -555, or -647 were applied for DAB staining or immunofluorescence staining. Images were acquired with a fluorescence microscope (AxioImager M2; Zeiss) or confocal microscope (LSM700; Carl Zeiss) equipped with a 40 × 1.25 NA objective lens (Plan-Apochromat; Carl Zeiss) and Zen 2009 (Carl Zeiss) acquisition and analysis software. For publication, the images were processed using Photoshop, with minimal adjustment to contrast or brightness applied to the entire images.

### Odorant-induced neuronal activation

Adult mice were individually housed for at least 1 week before assay. We added 20 μl of 1 μM limonene in mineral oil or vehicle control (mineral oil alone) to filter paper attached to a glass plate and placed it in the corner of the home cage for 15 min. Mice were anesthetized and perfused with 4% PFA 2 h later. Coronal sections (50 μm thickness) of the olfactory bulb and other brain regions were collected with a cryomicrotome. Immunohistochemistry with anti-C-FOS antibody (1:200; Cell Signaling) followed by DAPI staining was performed as described above. The numbers of C-FOS-positive cells in the regions of the olfactory system (Figs. 6 and 7) were then measured with ImageJ (NIH).

### Nissl staining

Sections were first attached to gelatin-coated glass slides. After rinsing with water, sections were stained with 0.1% cresyl violet solution (Sigma) in 1% acetic acid for 5 min, followed by rinsing with water, destaining with 70% EtOH, and then dehydration for mounting using Premount.

### Statistical analysis

Data collection and analysis in this report were conducted randomly and blind. All quantitative data are presented as means plus s.e.m. Graphs were plotted using GraphPad Prism 5.0 (GraphPad software). No statistical method was applied to evaluate the sample size, but our sample sizes are similar to those of previous publications [23, 59, 60]. Figure 1c and f, Fig. 6e and f, Fig. 7d and e, and Fig 8a and f were analyzed by unpaired *t* test. Figure 1d and e and Fig. 8b and c were analyzed by paired *t* test. Figure 1b was analyzed by two-way repeated measures (RM) ANOVA. *p* values of less than 0.05 were considered significant. Statistical analysis of MRI data was conducted as described [58] based on FDR. FDR < 0.1 was considered significant.

## Results

### Impairment of olfactory discrimination in *Tbr1*^*+/−*^ mice

To investigate whether *Tbr1* heterozygosity has any impact on olfaction, we evaluated olfactory sensation and discrimination in *Tbr1*^*+/−*^ mice. To set up the assay system, we first tested preferences for two distinct non-social odorants, limonene and 2-heptanol. After 2-day habituation to the presence of filter papers in their home cages, we separately spotted limonene and 2-heptanol onto two filter papers placed at two ends of the home cage (Fig. 1a, upper, preference test). The time spent sniffing limonene and 2-hepatonal was then measured. We found that both wild-type (WT) littermates and *Tbr1*^*+/−*^ mice spent similar amounts of time sniffing these two odorants (Fig. 1b; odorant effect: *F*_(1,9)_ = 2.437, *p* = 0.153, two-way RM ANOVA), suggesting that both WT and *Tbr1*^*+/−*^ mice can sense both limonene and 2-hepatonal and have no preference for either of them.

We then used these two odorants in six consecutive trials to examine the olfactory sensation and discrimination abilities of mice. Limonene and mineral oil (a control) were presented to mice in the first five trials with 15-min intervals (Fig. 1a, lower panel). The time spent sniffing limonene in the first trial indicated the olfactory sensation of mice. WT littermates and *Tbr1*^*+/−*^ mice spent comparable amounts of time sniffing limonene in trial 1 (Fig. 1c; *t*_(20)_ = 1.23, *p* = 0.2331, unpaired *t* test). Compared with mineral oil, both *Tbr1*^*+/−*^ mice and WT littermates spent significantly longer time sniffing limonene in trial 1 (Fig. 1d, trial 1; WT, *t*_(10)_ = 6.559, *p* < 0.0001; *Tbr1*^*+/−*^*, t*_(10)_ = 5.147, *p* = 0.0004, paired *t* test). The results suggested *Tbr1*^*+/−*^ mice exhibit normal olfactory sensation. The repetitive exposure to limonene in consecutive trials 2 to 5 habituated mice to limonene and adapted their olfactory responses to this odor (Fig. 1d). We found that time spent sniffing limonene rapidly decreased to levels comparable to those recorded for responses to mineral oil in both *Tbr1*^*+/−*^ mice and WT littermates during subsequent trials 2–5 (Fig. 1d; trial 2: WT, *t*_(10)_ = 1.762, *p* = 0.1085; *Tbr1*^*+/−*^*, t*_(10)_ = 0.5437, *p* = 0.5986, paired *t* test), indicating that habituation, i.e., olfactory adaptation, is also normal in *Tbr1*^*+/−*^ mice.

The olfactory discrimination ability of *Tbr1*^*+/−*^ mice was then investigated in trial 6, representing the dishabituation test. Limonene (the familiar odorant) and 2-heptanol (a novel odorant) were simultaneously presented in the home cages of mice during trial 6 (Fig. 1a, lower panel). All WT littermates spent significantly more time sniffing 2-heptanol (Fig. 1e; WT, *t*_(10)_ = 6.981, *p* = 0.001, paired *t* test), suggesting that WT mice were able to distinguish 2-heptanol from limonene. However, of the 11 *Tbr1*^*+/−*^ mice we assayed, only five animals spent more time sniffing 2-heptanol (Fig. 1e; Tbr1^+/−^, *t*_(10)_ = 2.109, *p* = 0.0611, paired *t* test). We then calculated an odor preference index by comparing the limonene and mineral oil data from trial 1 and the 2-heptanol and limonene data from trial 6 (see “Methods” section and Fig. 1f). We found that the preference indices for trial 1 were comparable between WT and *Tbr1*^*+/−*^ mice (Fig. 1f; *t*_(20)_ = 0.4123, *p* = 0.6845, unpaired *t* test), further supporting the conclusion that *Tbr1*^*+/−*^ mice have no defect in olfactory sensation. However, for trial 6, the preference indices of *Tbr1*^*+/−*^ mice were significantly lower than those of WT littermates (Fig. 1f; *t*_(20)_ = 2.981, *p* = 0.0074, unpaired *t* test). These results suggest that deletion of one allele of the *Tbr1* gene impairs olfactory discrimination but not olfactory sensation or adaptation.

### *Tbr1* expression in the olfactory system of mouse brains

**Fig. 2:**
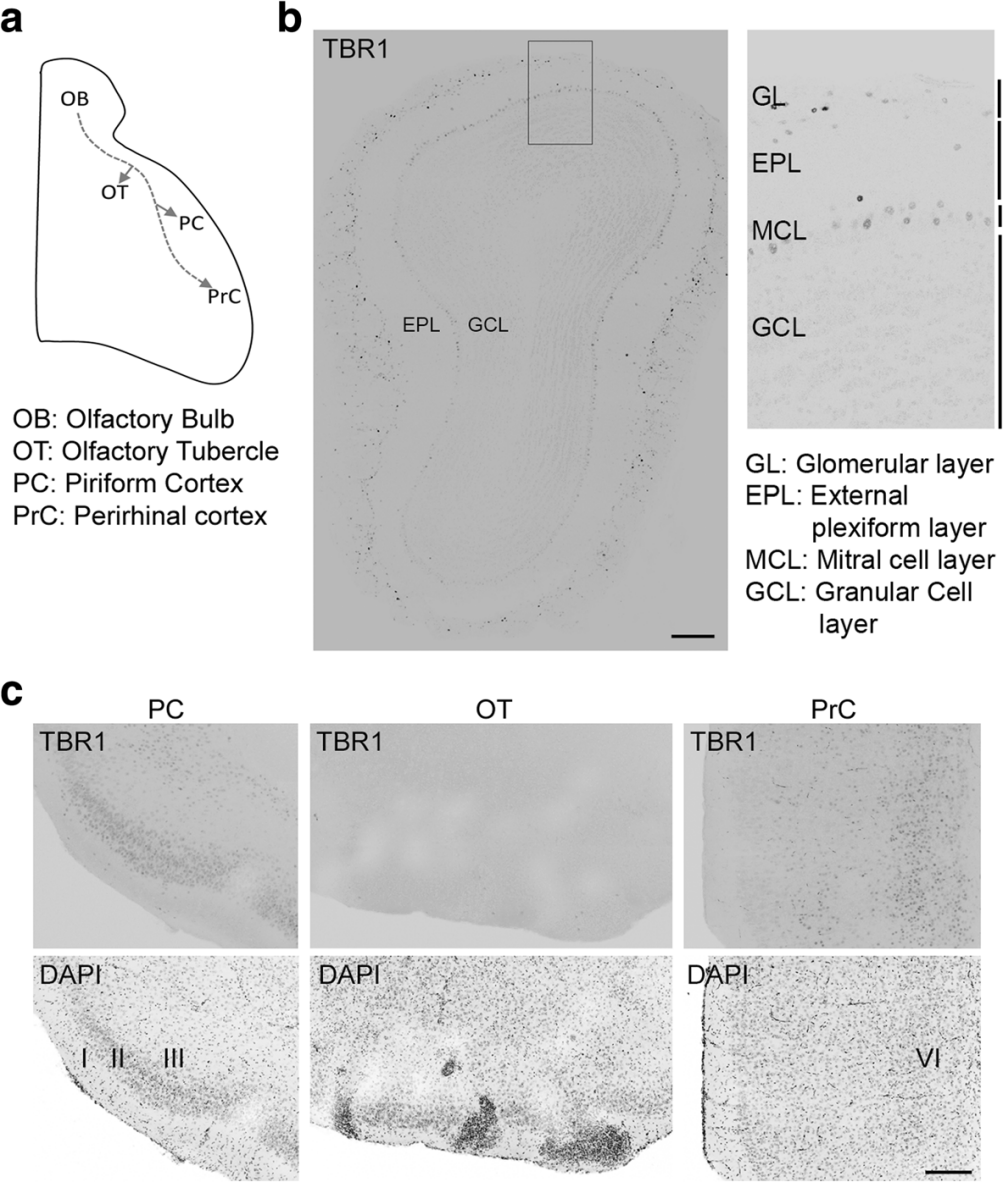
TBR1 expression pattern in the olfactory system of wild-type mouse brain. **a** Schematic of the four brain regions associated with olfaction. (**b**)–(**c**) Immunofluorescence staining using TBR1 antibody and DAPI counter-staining were performed to examine the expression of TBR1 in adult mouse brains. **b** TBR1 expression in the mitral cell layer and glomerular layer of the olfactory bulb. **c** TBR1 is expressed in the piriform and perirhinal cortices, but not in the olfactory tubercle. Scale bar 200 μm (**b**), (**c**)

To investigate how *Tbr1* haploinsufficiency regulates olfaction, we examined *Tbr1* expression in the olfactory system of WT mouse brains (Fig. 2a). Consistent with previous findings that TBR1 is expressed in mitral cells, tufted cells, and juxtaglomerular excitatory neurons of the olfactory bulb [26, 32, 61, 62], we also found that TBR1 was mainly expressed in the mitral cell layer and glomerular layer of the olfactory bulb in adult WT mice (Fig. 2b). In addition to the olfactory bulb, immunostaining also detected TBR1 expression in the piriform cortex (PC), mainly at the layer II projection neurons and in the perirhinal cortex (PrC), enriched at layer VI (Fig. 2c). However, there was no TBR1 signal in the olfactory tubercle (OT) (Fig. 2c). These immunostaining results indicate that TBR1 is expressed in several regions of the olfactory system of mouse brains.

### Alteration of the olfactory system in *Tbr1*^*+/−*^ mouse brains

**Fig. 3:**
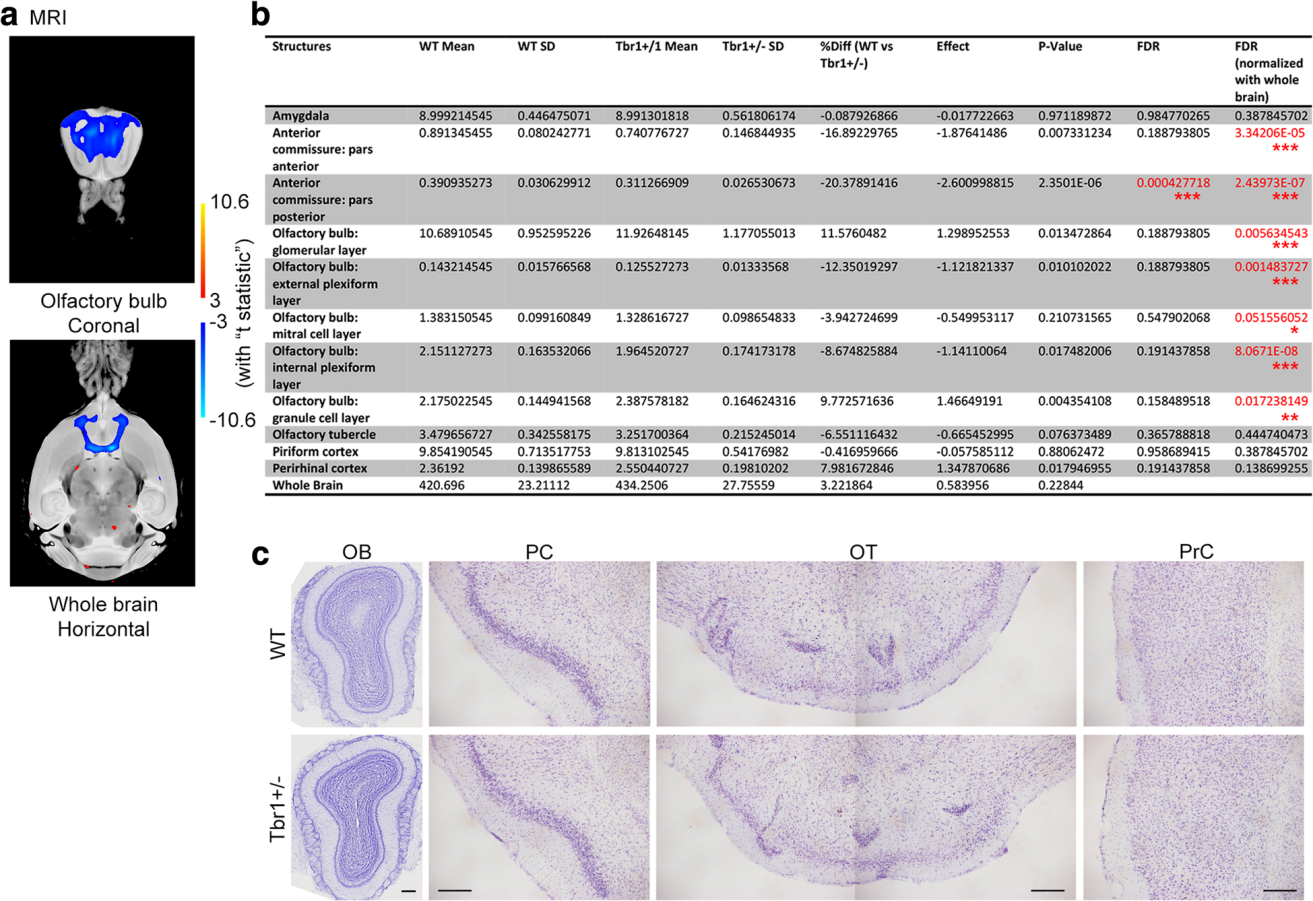
MRI reveals that *Tbr1*^+/−^ mice have a smaller anterior commissure and olfactory bulbs. **a** MRI brain images showing the reduced size of the olfactory bulbs and anterior commissure of *Tbr1*^+/−^ mice compared to WT littermates. Upper, coronal section of olfactory bulbs; lower, horizontal section. Pseudocolor indicates regions that are either enlarged or reduced in *Tbr1*^+/−^ mice. **b** Quantification of the sizes of brain regions associated with olfaction. Before normalization with whole-brain size, only the posterior part of the anterior commissure is different (red, FDR < 0.1). After normalization with whole-brain size, more regions exhibit differences (indicated in red). * FDR < 0.1; ** FDR < 0.05; *** FDR < 0.01. **c** Normal lamination and organization of the olfactory system in *Tbr1*^+/−^ mice, as revealed by Nissl stain. Scale bars 200 μm, (c)

We then investigated whether the deletion of an allele of the *Tbr1* gene alters anatomic or histological features of the olfactory system in mouse brains. We performed MRI to compare the size of the olfactory system of *Tbr1*^*+/−*^ mice and WT littermates. Consistent with previous histological analysis [23], our MRI results showed that the posterior part of the anterior commissure is the most sensitive region to *Tbr1* haploinsufficiency, even without normalization against whole-brain size (Fig. 3a and b). After normalizing against whole-brain size, the anterior part of the anterior commissure and the olfactory bulb (including the glomerular, external plexiform, mitral cell, internal plexiform, and granule cell layers) were smaller in *Tbr1*^*+/−*^ mice (Fig. 3a and b). However, the olfactory tubercle, piriform cortex, or perirhinal cortex were not affected by *Tbr1* haploinsufficiency (Fig. 3b). We then conducted Nissl staining to investigate whether histological features of the olfactory system were altered by *Tbr1* haploinsufficiency. We found that cellular organization and the laminar structure of the olfactory bulb, olfactory tubercle, piriform cortex, and perirhinal cortex were all normal in *Tbr1*^*+/−*^ mice (Fig. 3c). Thus, our MRI analysis and Nissl staining suggest that the size, but not structure, of the anterior commissure and olfactory bulb are particularly sensitive to *Tbr1* haploinsufficiency.

### Characterization of *Tbr1*^*+/−*^ olfactory bulbs using various markers

**Fig. 4:**
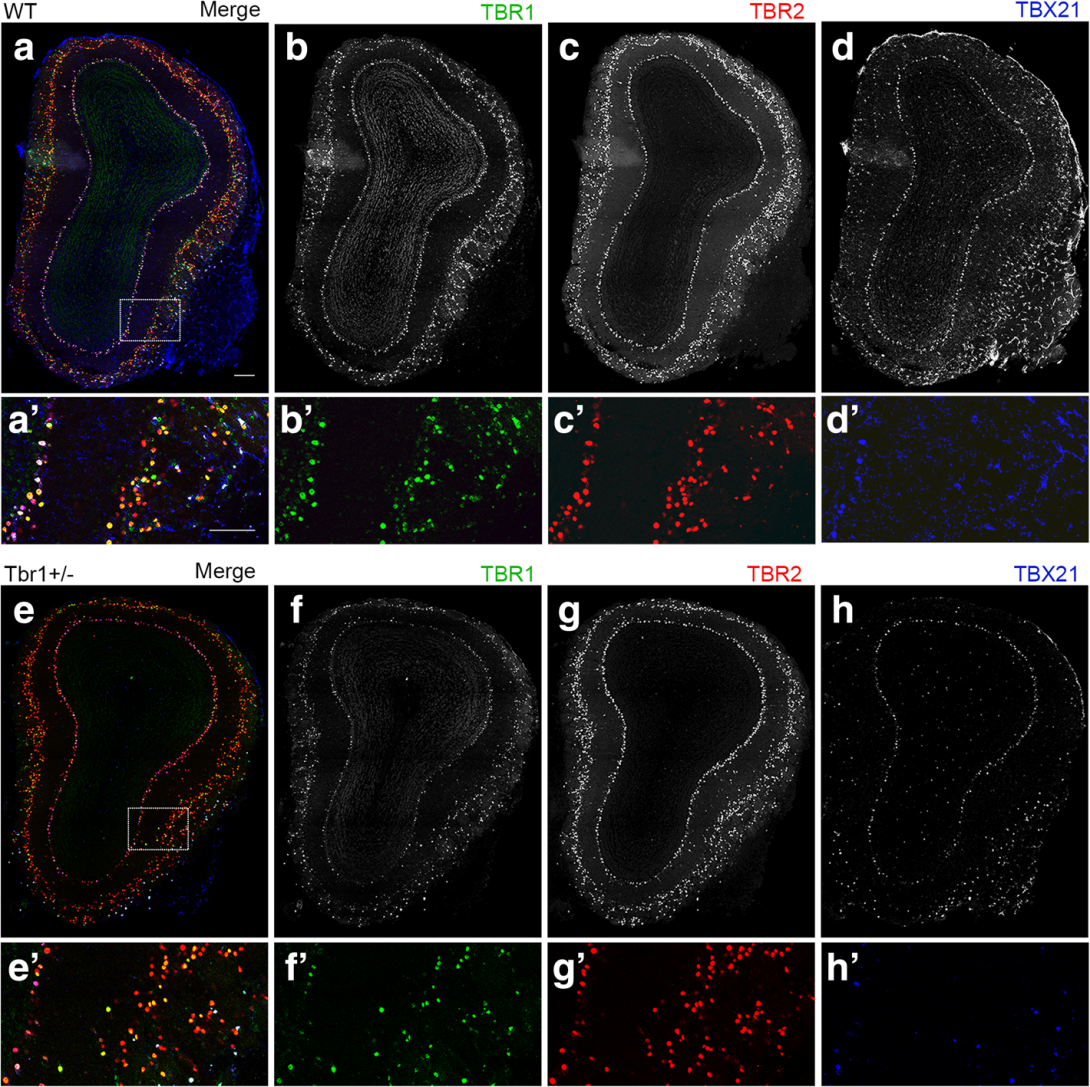
Expression of TBR1 subfamily members in *Tbr1*^*+/−*^ olfactory bulbs. Triple immunofluorescence labeling of TBR1, TBR2, and TBX21 was performed using adult WT littermates (**a**, **b**, **c**, **d**) and *Tbr1*^*+/−*^ mice (**e**, **f**, **g**, **h**). merged views (**a**, A′, **e**, E′); TBR1, green (**b**, B′, **f**, F′); TBR2, red (**c**, C′, **g**, G′); TBX21, blue (**d**, D′, **h**, H′). **a**–**h** whole olfactory bulb; A′ –H′ higher magnification of insets. Scale bars 200 μm, (**a**)–(**h**); 100 μm, (A′)–(H′)

We performed immunofluorescence staining with various markers to further characterize *Tbr1*^*+/−*^ olfactory bulbs. The first set of markers we used comprised members of the TBR1 subfamily of T-box transcription factors, including TBR1, TBR2 (T-brain-2, also known as Eomesodermin or EOMES), and TBX21 (also known as T-bet). Although TBR1 subfamily members are all expressed in mitral cells, tufted cells, and juxtaglomerular excitatory neurons of the olfactory bulb, only some of those cells express all three of these transcription factors [32]. Thus, differential expression of TBR1 subfamily members defines subpopulations of excitatory neurons in olfactory bulbs, though the biological functions of these different subpopulations are still unknown. In *Tbr2*^*−/−*^ neurons, TBR1 expression is upregulated, whereas TBX21 protein levels are reduced [32]. We wondered whether *Tbr1* haploinsufficiency also influences expression of other members of the TBR1 subfamily. We carried out triple immunostaining using TBR1, TBR2, and TBX21 antibodies to analyze adult olfactory bulbs. Our results revealed that TBR2:TBR1:TBX21 triple-positive mitral cells accounted for a sizeable proportion (~ one third) of all mitral cells in WT mice (Fig. 4, white nuclei in WT). Double-positive cells were also frequently found in WT olfactory bulbs (Fig. 4, yellow or purple nuclei in WT). In *Tbr1*^*+/−*^ olfactory bulbs, the general patterns of TBR1 subfamily-positive cells were similar to those of WT mice, but TBR2 seemed to be dominant and the number of triple-positive cells was reduced (Fig. 4, *Tbr1*^*+/−*^). Thus, the properties of projection neurons in olfactory bulbs are likely altered by *Tbr1* deficiency.

**Fig. 5:**
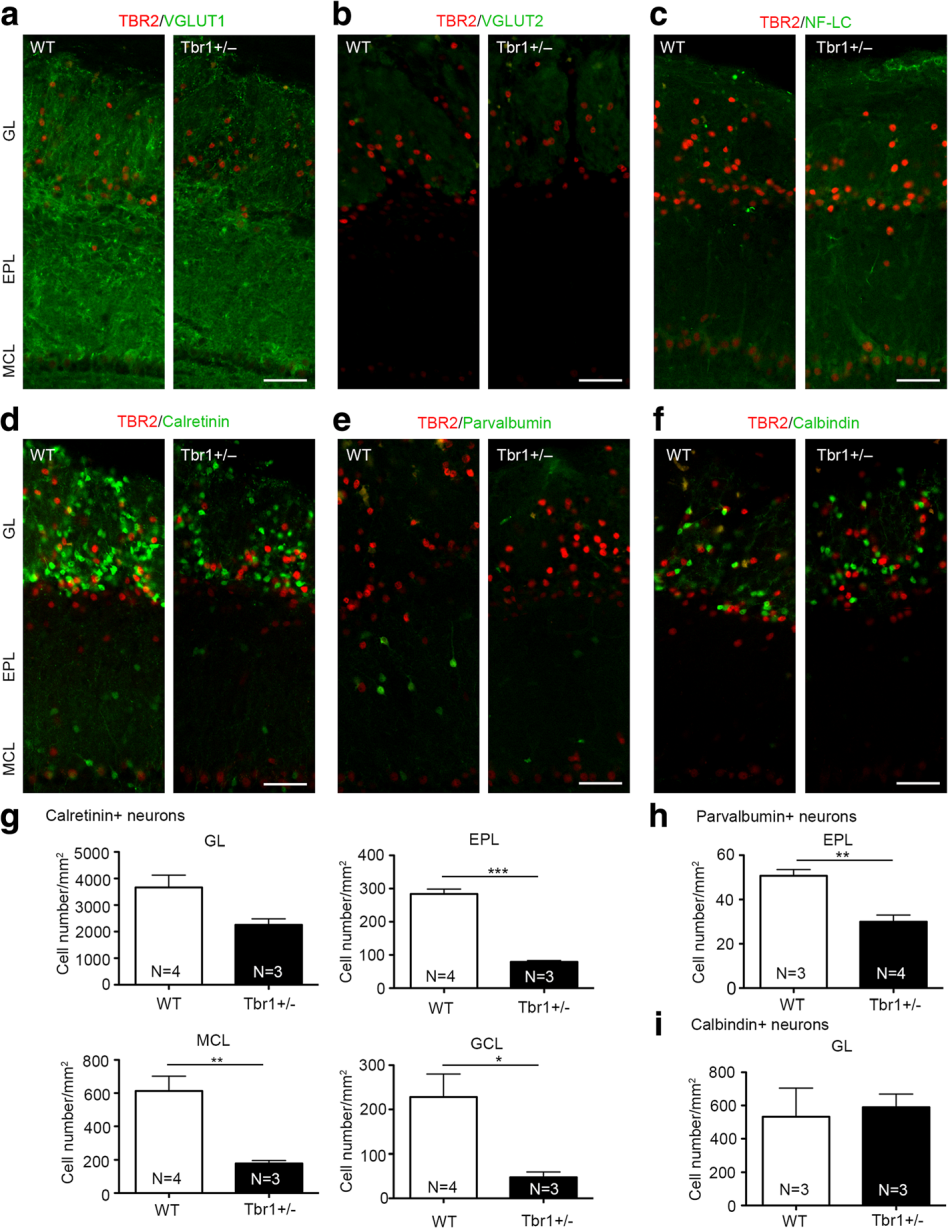
Reduction of inhibitory interneurons and morphological alteration of the mitral cell layer in *Tbr1*^*+/−*^ olfactory bulbs. Double immunostaining of TBR2 and **a** VGLUT1, **b** VGLUT2, **c** Neurofilament-light chain (NF-LC), **d** Calretinin, **e** Parvalbumin, and **f** Calbindin was performed to compare olfactory bulbs of *Tbr1*^*+/−*^ and WT mice. **g** Quantification of Calretinin^+^ interneurons at the glomerular layer (GL), external plexiform layer (EPL), mitral cell layer (MCL), and granular cell layer (GCL). **h** Quantification of parvalbumin^+^ interneurons at the external plexiform layer (EPL). **i** Quantification of calbindin^+^ interneurons at the glomerular layer (GL). Data represent mean plus SEM and the results of individual mice are shown. *p < 0.05; **p < 0.01; ***p < 0.001. Scale bars: 50 μm

A previous study indicated that *Tbr2* deletion alters expression of vesicular glutamate transporters (VGLUTs) in mitral and tufted cells, and influences dendrodendritic synapses in the external plexiform layer of olfactory bulbs [32]. To investigate whether *Tbr1* haploinsufficiency influences the expression of VGLUTs, we performed immunostaining using antibodies against VGLUT1 and VGLUT2. We found that ratios of VGLUT1 and VGLUT2 signals in the glomerular to external plexiform layers were not altered in *Tbr1*^*+/−*^ olfactory bulbs compared to those of WT littermates (Fig. 5a and b, Additional file 1: Figure S1). Thus, unlike *Tbr2* deficiency [32], *Tbr1* haploinsufficiency does not result in a shift from VGLUT1 to VGLUT2. Based on immunostaining with neurofilament antibody, *Tbr2*^*−/−*^ mitral cells exhibit thinner and more disorganized dendrites compared with those of WT cells [32]. In contrast to the outcome of *Tbr2* deletion, the dendrites of *Tbr1*^*+/−*^ mitral cells became thicker (Fig. 5c, Additional file 1: Figure S1). Thus, TBR1 and TBR2 play differential roles in controlling synapse transmission and dendritic organization of mitral cells.

We then used calretinin, parvalbumin, and calbindin antibodies as markers to monitor interneurons in olfactory bulbs. Calretinin^+^ neurons were widely distributed at different layers, including the glomerular layer (GL), external plexiform layer (EPL), mitral cell layer (MCL), and granular cell layer (GCL) (Fig. 5d, Additional file 1: Figure S1). All these layers showed fewer calretinin^+^ neurons in *Tbr1*^*+/−*^ olfactory bulbs compared to WT littermates, though only the differences for EPL, MCL, and GCL were significant (Fig. 5g; GL, *t*_(5)_ = 2.429, *p* = 0.0595; EPL, *t*_(5)_ = 11.55, *p* = < 0.0001; MCL, *t*_(5)_ = 4.065, *p* = 0.0097; GCL, *t*_(5)_ = 2.898, *p* = 0.0339, unpaired *t* test). Parvalbumin^+^ interneurons were enriched at the EPL (Fig. 5e, Additional file 1: Figure S1) and had a lower cell density in *Tbr1*^*+/−*^ olfactory bulbs relative to WT littermates (Fig. 5h; *t*_(5)_ = 4.88, *p* = 0.0046, unpaired *t* test). Calbindin^+^ interneurons were present in the GL (Fig. 5f, Additional file 1: Figure S1) but there was no significant difference between *Tbr1*^*+/−*^ mice and WT littermates (Fig. 5i; *t*_(4)_ = 0.3014, *p* = 0.7782, unpaired *t* test). Since TBR1 is not expressed in interneurons, the reduction of calretinin^+^ and parvalbumin^+^ interneurons is a non-cell autonomous effect. Only parvalbumin signals were obviously altered in *Tbr2*^*−/−*^ mice [32]. Therefore, *Tbr1* and *Tbr2* exhibit differing non-cell-autonomous effects on interneurons.

Our immunostaining results using various markers suggest that *Tbr1* heterozygosity likely influences projection neurons and alters inhibitory interneurons. These defects are specific for *Tbr1* haploinsufficiency and cannot be compensated for by the presence of *Tbr2*.

### Reduced neuronal activation in *Tbr1*^*+/−*^ mouse brains

**Fig. 6:**
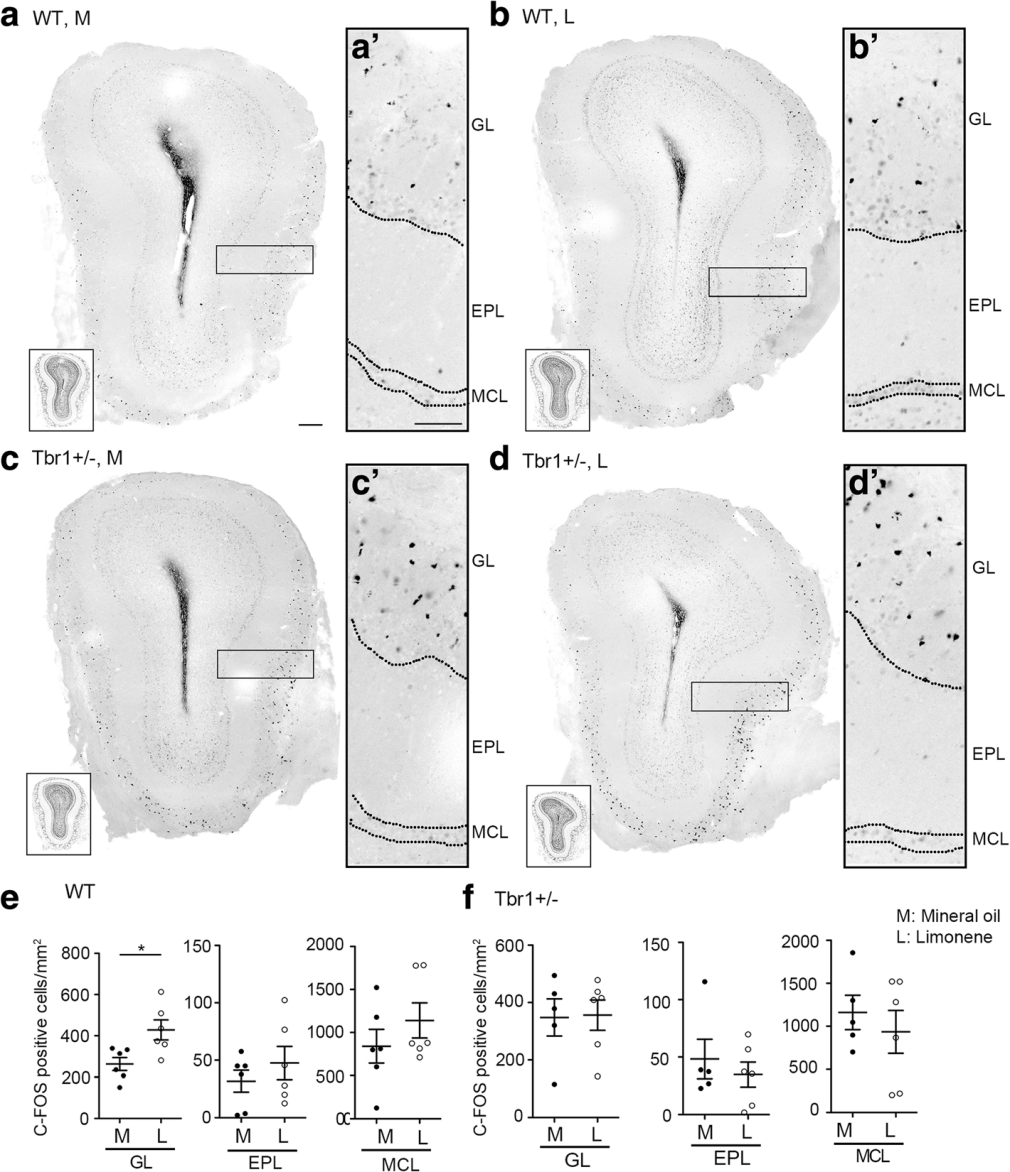
Increased neuronal activation in the glomerular layer of WT littermates but not *Tbr1*^*+/−*^ mice. **a**-**d** Two hours after exposure to limonene or mineral oil, *Tbr1*^*+/−*^ mice and WT littermates were subjected to C-FOS staining to monitor neuronal activation. **a**’–**d**’ High magnification images of insets in (**a**)–(**d**). **e**–**f** Quantification of C-FOS-positive cell number in the glomerular layer (GL), external plexiform layer (EPL), and mitral cell layer (MCL). Data represent mean plus SEM and the results of individual mice are shown. * *p* < 0.05. Scale bars 200 μm (original images); 100 μm (enlarged images)

We then investigated whether neuronal activation in the olfactory system is altered by *Tbr1* haploinsufficiency, resulting in impairment of olfactory responses. Two hours after exposure to limonene for 15 min, we examined C-FOS expression by immunostaining to monitor neuronal activation (Fig. 6a, b, c, and d). Compared with a mineral oil control, limonene stimulation resulted in more C-FOS-positive cells in the GL of WT mice (Fig. 6e and f; GL, WT, *t*_(10)_ = 2.863, *p* = 0.0169, unpaired *t* test) but not in *Tbr1*^*+/−*^ mice (Fig. 6e and f; GL, *Tbr1*^*+/−*^, *t*_(9)_ = 0.09979, *p* = 0.9227, unpaired *t* test). In both the EPL and the MCL, we did not observe any change in C-FOS cell number in WT littermates or *Tbr1*^*+/−*^ mice (Fig. 6e and f; EPL: WT, *t*_(10)_ = 0.911, *p* = 0.3838; *Tbr1*^*+/−*^, *t*_(9)_ = 0.6923, *p* = 0.5062; MCL: WT, *t*_(10)_ = 1.061, *p* = 0.3138; *Tbr1*^*+/−*^, *t*_(9)_ = 0.6838, *p* = 0.511, unpaired *t* test). Thus, only the GL exhibits lower neuronal activation upon odor stimulation in *Tbr1*^*+/−*^ olfactory bulbs.

**Fig. 7:**
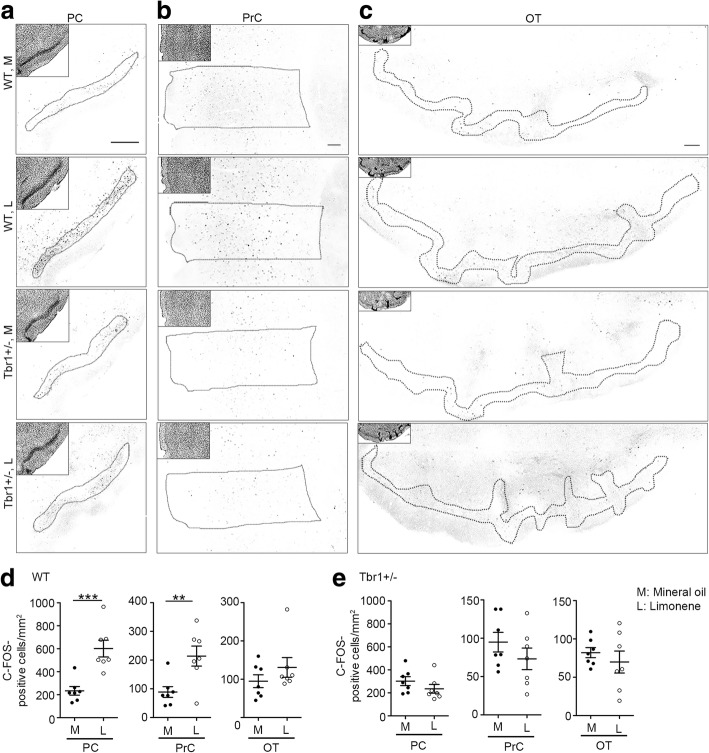
Impaired neuronal activation in the piriform and perirhinal cortices due to *Tbr1* haploinsufficiency. **a**–**c** Two hours after exposure to limonene (L) or mineral oil (M), mouse brains were harvested for C-FOS staining. Insets are DAPI images to outline the structure of different brain regions (the piriform (PC) and perirhinal (PrC) cortices and the olfactory tubercle (OT)). **d**–**e**. Quantitative data showing the numbers of C-FOS-positive cells in WT (**d**) and *Tbr1*^*+/−*^ mice (e). Data represent mean plus SEM and the results of individual mice are shown. **p < 0.01, ***p < 0.001. Scale bars 200 μm (**a**); 100 μm (**b**); 100 μm (**c**)

In the upper olfactory system of WT mice, the numbers of C-FOS-positive cells in both the anterior piriform and perirhinal cortices, but not olfactory tubercles, was increased upon limonene stimulation compared with a mineral oil control (Fig. 7d and e; WT: anterior piriform, *t*_(12)_ = 4.486, *p* = 0.0007; perirhinal, *t*_(12)_ = 3.17, *p* = 0.0081; olfactory tubercle, *t*_(12)_ = 1.172, *p* = 0.2641, unpaired *t* test). In *Tbr1+/−* mice, neither the piriform and perirhinal cortices nor olfactory tubercles exhibited increases of C-FOS-positive cell numbers upon comparing results for limonene with mineral oil control (Fig. 7e; *Tbr1*^*+/−*^: anterior piriform, *t*_(12)_ = 1.236, *p* = 0.2401; perirhinal, *t*_(12)_ = 1.148, *p* = 0.2734; olfactory tubercles, *t*_(12)_ = 0.7735, *p* = 0.4542, unpaired *t* test). Thus, the defects of neuronal activation in response to odor stimulation primarily lie in the glomerular layer of olfactory bulbs and the piriform and perirhinal cortices of *Tbr1*^*+/−*^ mice.

### D-cycloserine has a beneficial effect on olfactory discrimination of *Tbr1*^*+/−*^ mice

**Fig. 8:**
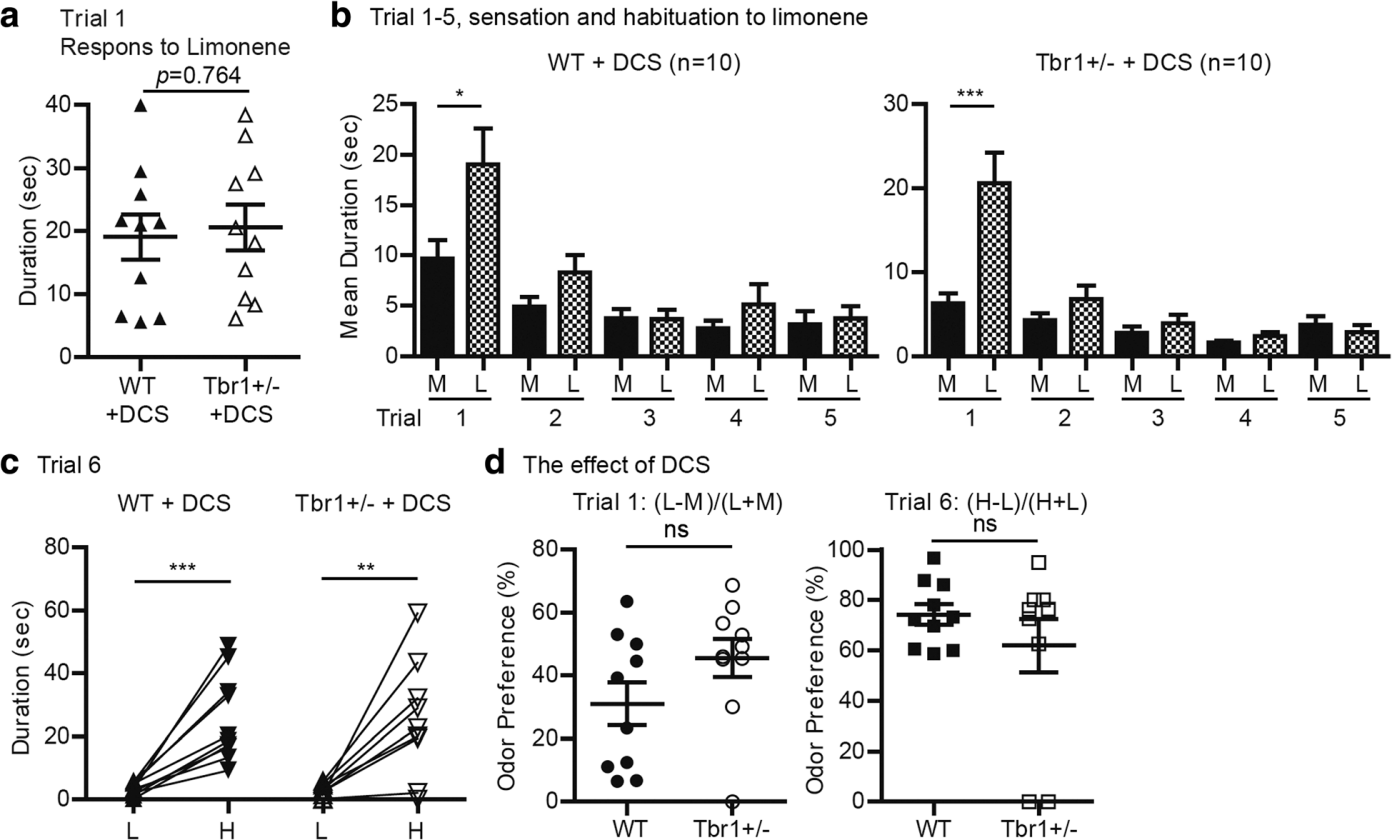
Rescue effect of D-cycloserine on olfactory discrimination in *Tbr1*^*+/−*^ mice. **a**–**b** Thirty minutes after D-cycloserine (DCS) treatment, mice were subjected to the sensation-habituation-dishabituation test, as indicated in the lower panel of Fig. 1a. **a** Time spent sniffing limonene in the first trial and **b** olfactory habituation are comparable between WT and *Tbr1*^+/−^ mice. **c**–**d** D-cycloserine improves olfactory discrimination of *Tbr1*^*+/−*^ mice in trial 6. Data represent mean plus SEM and the results of individual mice are shown. *p < 0.05; **p < 0.01; ***p < 0.001

Our previous study indicated that *Tbr1* haploinsufficiency impairs axonal connectivity and neuronal activation of amygdalar neurons [23]. Systemic administration or local infusion of D-cycloserine into amygdalae effectively ameliorates impaired neuronal activation of amygdalae and associated behavioral deficits in social interaction, cognitive flexibility, and memory [23]. Since neuronal activation in the glomerular layer of the olfactory bulb and its piriform and perirhinal cortices was impaired in *Tbr1*^*+/−*^ mice upon odor stimulation (Fig. 7), we wondered if the olfactory defects of *Tbr1*^*+/−*^ mice could be improved by D-cycloserine treatment. To test this possibility, we intraperitoneally injected D-cycloserine into both WT and *Tbr1*^*+/−*^ mice 30 min before undergoing an olfactory discrimination test. Similar to the results without D-cycloserine treatment (Fig. 1), both WT and *Tbr1*^*+/−*^ mice behaved comparably in terms of olfactory sensation (Fig. 8a; *t*_(18)_ = 0.3053, *p* = 0.7636, unpaired *t* test) and adaptation to limonene (Fig. 8b). Importantly, in the discrimination test (trial 6), both *Tbr1*^*+/−*^ and WT mice spent significantly more time sniffing 2-heptanol, i.e., the novel odorant (Fig. 8c; WT, *t*_(9)_ = 5.479, *p* = 0.0004; Tbr1^+/−^, *t*_(9)_ = 3.517, *p* = 0.0065; paired *t* test). Odor preferences of *Tbr1*^*+/−*^ mice in both trials 1 and 6 were also comparable to those of WT mice (Fig. 8d; trial 1, *t*_(18)_ = 1.601, *p* = 0.1269; trial 6, *t*_(18)_ = 1.074, *p* = 0.2970; unpaired *t* test). These results suggest that, similar to its effect on amygdalar deficits caused by *Tbr1* haploinsufficiency, increased neuronal activation by D-cycloserine ameliorates impaired olfactory discrimination in *Tbr1* mutant mice.

## Discussion

Previous reports have shown that *Tbr1*^*+/−*^ mice, a mouse model for autism, exhibit reduced social interaction, cognitive inflexibility, and defective associative memory [23]. In this report, we further show that olfactory discrimination to non-social odors is impaired in *Tbr1*^*+/−*^ mice. The olfactory bulbs and anterior commissure are smaller in *Tbr1*^*+/−*^ mice. Although we observed a reduced population of calretinin^+^ and parvalbumin^+^ interneurons in *Tbr1*^*+/−*^ olfactory bulbs, neuronal activation in the glomerular layer is still reduced in *Tbr1*^*+/−*^ mice upon odorant stimulation, suggesting abnormal local neural circuits in olfactory bulbs of *Tbr1*^*+/−*^ mice. In the upper region of the olfactory system, the sizes of piriform and perirhinal cortices are not changed by *Tbr1* haploinsufficiency. However, neuronal activation of these two regions is not induced in response to odorant stimulation in *Tbr1*^*+/−*^ mice, suggesting that the neuronal circuit from the olfactory bulbs to the upper olfactory system is impaired by *Tbr1* haploinsufficiency. Moreover, D-cycloserine treatment to increase neuronal activity rescues the defect of olfactory discrimination in *Tbr1*^*+/−*^ mice, echoing a previous study showing that circuit defects in *Tbr1* mutant mice lead to lower neuronal activity and abnormal behavior [23].

Olfactory dysfunction has been reported for patients with ASD [34–39, 41, 42]. An alcohol sniff test, a standardized clinical evaluation of olfactory detection, was previously used to show the enhanced olfactory sensitivity of patients with ASD [37]. This test involves using antiseptic swabs of isopropyl alcohol (70% vol.) as stimuli to minimize trigeminal effects and other cognitive demands. It showed that some patients with ASD have a lower threshold for detecting isopropyl alcohol [37]. In other studies using 1-butanol and a variety of food odorants, patients with ASD or Asperger’s syndrome exhibited normal sensing ability but diminished ability to identify different odors, reflecting impaired olfactory discrimination [34, 35, 39]. These studies indicate diverse olfactory phenotypes among patients. However, why and how olfaction is impaired in ASD is unclear. In this report, *Tbr1*^*+/−*^ mice exhibit normal olfactory sensation but impaired olfactory discrimination to non-social odors, recapitulating one type of olfactory defect observed in ASD. Importantly, our study revealed defective neural circuits and reduced neuronal activity in the olfactory system caused by *Tbr1* deficiency. By increasing neuronal activity through D-cylcoserine treatment, we could improve the olfactory discrimination ability of *Tbr1*^*+/−*^ mice. This outcome echoes our previous finding that reduced social interaction, cognitive inflexibility, and impaired associative memory of *Tbr1*^*+/−*^ mice are ameliorated by D-cycloserine treatment [23]. Thus, our evidence supports the hypothesis that *Tbr1* deficiency alters neural circuits (by changing axonal projection and neuronal activation), resulting in autism-like behaviors, and that these defects can be improved by increasing neuronal activity via D-cycloserine treatment. For ASD patients characterized by NMDAR hypoactivation (such as arises from *TBR1* deficiency), D-cycloserine or other compounds with a similar pharmacological effect might represent a potential medicine for ameliorating patient symptoms.

In terms of animal studies, mutations of *Fragile X mental retardation 1* (*dFmr1*) in *Drosophila* result in lower activity of GABAergic interneurons and thus reduce lateral inhibition of excitatory projection neurons in olfactory glomeruli, resulting in higher activity of projection neurons and consequently impairing odor selectivity [11]. In our current study, we also found that cell numbers of calretinin^+^ and parvalbumin^+^ interneurons are reduced in *Tbr1*^*+/−*^ olfactory bulbs, particularly in the EPL, MCL, and GCL (Fig. 5). Accordingly, localized inhibition from interneurons is likely to be reduced. It has been shown that mitral cells, the major projection neurons sending output signals to higher brain regions, reciprocally innervate local interneurons in olfactory bulbs [63]. Local interneurons that receive the excitatory signals from mitral cells can increase their activity to inhibit neighboring mitral cells, thereby reducing noise and sharpening the responses of mitral cells, which has been suggested to be a critical process in olfactory discrimination to identify odors and map complex dimensional odors into dynamic ensembles of neuronal activity [64–68]. In addition, the anterior part of the anterior commissure that provides contralateral inhibition between the two olfactory bulbs of the two brain hemispheres is smaller in *Tbr1*^*+/−*^ mice (Fig. 3a and b). Thus, olfactory processing within and between two olfactory bulbs is likely defective in *Tbr1*^*+/−*^ mice, although detailed electrophysiological recording will be required to further investigate this possibility. Based on our behavioral assays, these defects do not have an impact on olfactory sensation but specifically impair olfactory discrimination, consistent with the idea that local inhibition refines olfactory discrimination [11].

In olfactory bulbs, *Tbr1* haploinsufficiency only alters neuronal activity of the GL in olfactory bulbs. In the upper olfactory system, activation of piriform and perirhinal cortices is also impaired. Together, these findings suggest dysregulation of the neural circuits in the olfactory system, leading to impaired olfactory discrimination. However, it is still unclear why only neuronal activity of the GL in *Tbr1*^*+/−*^ olfactory bulbs is reduced. Since mitral cells also form reciprocal connectivity with neurons at the GL and mitral cells in *Tbr1*^*+/−*^ olfactory bulbs might not receive precise regulation from local interneurons at the EPL and MCL (see previous paragraph), mitral cells in *Tbr1*^*+/−*^ olfactory bulbs might not appropriately activate local neuronal activity at the GL. Consequently, neuronal activation in the GL of *Tbr1*^*+/−*^ mice is reduced. Certainly, this explanation is overly simplistic, as olfactory systems exhibit such highly complex local microcircuits with diverse reciprocal connectivity and feed-forward and -backward regulation. More detailed analysis is required to fully understand the circuit defects we observed. Nevertheless, our results echo that since olfaction relies on precise and complex neural circuits, it is extremely sensitive to circuit deficits such as those arising from ASD.

All three members of the TBR1 subfamily, i.e., TBR1, TBR2, and TBR21, are expressed in mitral cells, tufted cells, and juxtaglomerular excitatory neurons [32]. Based on mouse model studies, the functions of these three subfamily members in olfactory bulbs are obviously different from each other. Although TBR1 protein levels are upregulated in *Tbr2* conditional knockout mice, expression levels of VGluT1 and VGluT2 are reversed [32], suggesting that TBR1 upregulation does not compensate for *Tbr2* deficiency in VGluT1 and VGluT2 expression. Conversely, although *Tbr1* haploinsufficiency results in impaired olfactory discrimination, the ratio of VGLUT1 and VGLUT2 expression in glomerular and external plexiform layers is not obviously altered. It is interesting to note that in the cerebral cortex, TBR1 and TBR2 are expressed sequentially during development of projection neurons. TBR1 is specific for postmitotic neurons [24], whereas TBR2 is transiently expressed in the subventricular zone directly before TBR1 is expressed [69]. Thus, the functions of TBR1 and TBR2 in the cerebral cortex are also distinct. Since TBR1, TBR2, and TBX21 have highly homologous T-box domains, i.e., the DNA-binding domain located in the central region of the proteins [61], they likely bind the same DNA sequence. The distinct functions of TBR1, TBR2, and TBX21 are thus more likely to be mediated by the N- and C-terminal regions, which share less similarity in their amino acid sequences [61]. It will be interesting to investigate the molecular functions of the TBR1 subfamily in the future, results of which will further elucidate the regulatory mechanisms of the TBR1 subfamily in neurons. Understanding more about TBR1 and the relationship between TBR1 and other T-Box proteins may provide further information about the molecular etiology of TBR1-related ASD.

## Conclusion

Using *Tbr1*^*+/−*^ mice, we have demonstrated that TBR1 controls the circuits and activity of the olfactory system in mice. Olfactory discrimination but not olfactory sensation is specifically affected by *Tbr1* haploinsufficiency. Importantly, similar to amygdala-dependent behaviors, the olfactory deficiency exhibited by *Tbr1*^*+/−*^ mice can be ameliorated by increasing neuronal activity via D-cycloserine treatment. Our study suggests that increased neuronal activity can improve multiple autism-like behaviors in *Tbr1*^*+/−*^ mice, confirming that impaired neural circuits and activity are general features caused by *Tbr1* deficiency.

## Additional file

Additional file 1: **Figure S1.** Entire images of olfactory bulbs shown in Fig. 5. Double immunostaining of TBR2 and (A) VGLUT1, (B) VGLUT2, (C) Neurofilament-light chain (NF-LC), (D) Calretinin, (E) Parvalbumin, and (F) Calbindin was performed to compare *Tbr1+/−* and WT olfactory bulbs. Black and white images in the bottom-left corners of each entire image are DAPI staining results. Scale bars: 200 μm. (PDF 797 kb)

## Abbreviations

ASD: Autism spectrum disorders
EPL: External plexiform layer
GCL: Granule cell layers
GL: Glomerular layer
Grin2b: Glutamate ionotropic receptor NMDA type subunit
IPL: Internal plexiform layer
MCL: Mitral cell layer
NMDAR: N-methyl-D-aspartate receptor
OT: Olfactory tubercle
PC: Piriform cortex
PrC: Perirhinal cortex
Tbr1: T-brain-1
Tbr2: T-brain-2 or Eomes
Tbx21: T-box-21 or T-bet
VGLUT1: Vesicular glutamate transporter 1
VGLUT2: Vesicular glutamate transporter 2
