# Supplementary material for: Haploinsufficiency of autism causative gene Tbr1 impairs olfactory discrimination and neuronal activation of the olfactory system in mice
Source: Mol Autism. 2019 Feb 11;10:5. doi: 10.1186/s13229-019-0257-5 (PMC6371489; doi:10.1186/s13229-019-0257-5)
Supplement: Supplementary file 1 — Figure S1. Entire images of olfactory bulbs shown in Fig. 5. Double immunostaining of TBR2 and (A) VGLUT1, (B) VGLUT2, (C) Neurofilament-light chain (NF-LC), (D) Calretinin, (E) Parvalbumin, and (F) Calbindin was performed to compare Tbr1+/− and WT olfactory bulbs. Black and white images in the bottom-left corners of each entire image are DAPI staining results. Scale bars: 200 μm. (PDF 797 kb) [file 13229_2019_257_MOESM1_ESM.pdf]

Supplementary Figure and Figure Legend (by Huang, Yen et al.)

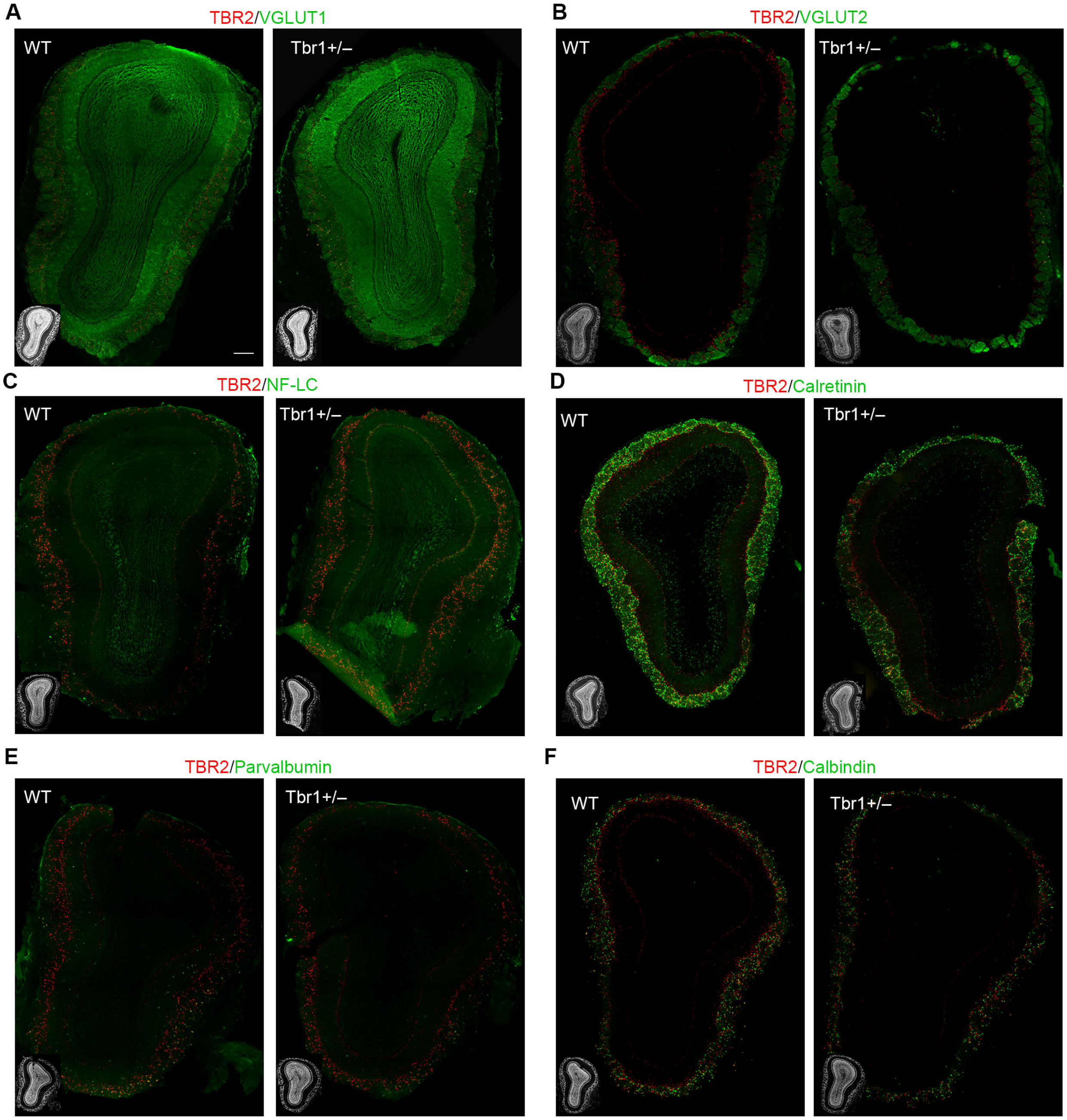

Figure S1. Entire images of olfactory bulbs shown in Figure 5.

Double immunostaining of TBR2 and (A) VGLUT1, (B) VGLUT2, (C) Neurofilament-light chain (NF-LC), (D) Calretinin, (E) Parvalbumin, and (F) Calbindin was performed to compare *Tbr1*$^{+/-}$ and WT olfactory bulbs. Black and white images in the bottom-left corners of each entire image are DAPI staining results. Scale bars: 200 μm.
